# Mental Health First Aid guidelines for helping a suicidal person: a Delphi consensus study in Japan

**DOI:** 10.1186/1752-4458-5-12

**Published:** 2011-05-19

**Authors:** Erminia Colucci, Claire M Kelly, Harry Minas, Anthony F Jorm, Yuriko Suzuki

**Affiliations:** 1 Centre for International Mental Health, Melbourne School of Population Health, The University of Melbourne, Parkville, Victoria 3010, Australia; 2 Mental Health First Aid Training and Research Program, Orygen Youth Health Research Centre, Centre for Youth Mental Health, The University of Melbourne, Parkville, Victoria 3052, Australia; 3 Department of Adult Mental Health, National Institute of Mental Health, NCNP, Kodaira, Tokyo, Japan

## Abstract

**Background:**

This study aimed to develop guidelines for how a member of the Japanese public should provide mental health first aid to a person who is suicidal.

**Methods:**

The guidelines were produced by developing a questionnaire containing possible first aid actions and asking an expert panel of 32 Japanese mental health professionals to rate whether each action should be included in the guidelines. The content of the questionnaire was based on a systematic search of the relevant evidence and claims made by authors of consumer and carer guides and websites. The panel members were asked to complete the questionnaire by web survey. Three rounds of the rating were carried and, at the end of each round, items that reached the consensus criterion were selected for inclusion in the guidelines. During the first round, panel members were also asked to suggest any additional actions that were not covered in the original questionnaire (to include items that are relevant to local cultural circumstances, values, and social norms). Responses to these open-ended questions were used to generate new items.

**Results:**

The output from the Delphi process was a set of agreed upon action statements. The Delphi process started with 138 statements, 38 new items were written based on suggestions from panel members and, of these 176 items, 56 met the consensus criterion. These statements were used to develop the guidelines appended to this article.

**Conclusions:**

There are a number of actions that are considered to be useful for members of the Japanese public when they encounter someone who is experiencing suicidal thoughts or engaging in suicidal behaviour. Although the guidelines are designed for members of the public, they may also be helpful to health professionals working in health and welfare settings who do not have clinical mental health training.

## Background

As reported by WHO [1], suicide is a huge but largely preventable public health problem, causing almost half of all violent deaths and resulting in one million fatalities every year (almost 3000 every day) worldwide, as well as economic costs in the billions of dollars. Estimates suggest fatalities could rise to 1.5 million by 2020 [2]. For every person who dies by suicide, 20 or more make a suicide attempt, resulting in injury, hospitalization, and emotional and mental trauma, although no reliable data are available on the full extent of suicide attempts [1,2].

In Japan, the annual number of suicides has been over 30,000, since a drastic increase in 1998, and suicide rates have been over 25.0 per 100,000 people, which is above the rate in other developed nations [3]. Suicide rates are particularly high among older men in rural areas, and are rapidly growing among middle aged men in urban areas [4]. As a counter-measure to this situation, the "Basic Act on Suicide Prevention" was issued in 2006, which emphasises building the capacity of gatekeepers to recognise and respond appropriately when a person is at risk of suicide [5].

For every suicide death, there are scores of family and friends whose lives are devastated emotionally, socially and economically [2]. For family members and friends affected by suicide or attempted suicide, the emotional impact may last for many years [1]. Also when the suicide attempt does not result in death, this act will have several consequences (emotional, physical, social and so on) for the individual as well as family, friends and others.

A member of the community who is close to the suicidal person, such as a friend, family member, co-worker or classmate, is likely to be the first person to notice suicide warning signs. However, few have the knowledge and skills required to recognize the imminent risk of suicide and to assist in preventing suicide. If members of the public were provided with simple and practical guidelines on how they should provide first aid to a person who is suicidal (i.e. has expressed suicidal thoughts or intent, whether overt or covert, or has taken action toward making a suicide attempt), they might be able to help a suicidal individual to seek professional help or decide against suicide. Such guidelines could be applied in training courses for the public, as for other first aid training courses.

First aid training is widespread throughout the world, giving members of the public skills to help an injured person before medical help arrives. There are many organizations offering first aid training, but the first aid practices taught in these courses generally conform to national guidelines. While first aid training is common, it generally ignores mental health crises, such as how to assist a suicidal person. Nevertheless, there have been efforts to develop training for the public that does cover these issues, such as Applied Suicide Intervention Skills Training (ASIST) [6] and Mental Health First Aid (MHFA) training [7]. In Japan, a brief and structured training course for medical residents on how to manage a suicidal patient has been developed and implemented by modifying the depression module of the MHFA program. The feasibility and preliminary effectiveness of the training were demonstrated, although some limitations were also highlighted [8]. The program is currently being extended to other human service workers who may encounter suicidal persons in their daily practice.

Unfortunately, there is limited evidence to guide the content of such trainings. While randomized controlled trials provide the highest standard of evidence, it is not feasible or ethical to carry out such trials to evaluate specific suicide first aid strategies. In the absence of high quality evidence, the best option for developing guidelines is expert consensus [9]. There are formal methods for assessing expert consensus that have been used in several areas of health research. One of the most commonly used consensus method is the *Delphi process *[10]. There are many variants but all involve a group of experts making private ratings of level of agreement with a series of statements, feedback to the group of a statistical summary of the ratings, and then another round of rating. Delphi group members do not meet, so it is possible to do studies using mail or the Internet. The output from the process is statements for which there is substantial consensus in ratings. The Delphi method has been used in health research since the mid-1970s [11]. The Delphi method has been used to develop suicide first aid guidelines for developed English-speaking countries [12], as well as mental health first aid guidelines for non-suicidal self injury [13], panic attacks [14], psychosis [15], depression [16], eating disorders [17], problem drinking [18], problem drug use [19] and traumatic events [20]. However, guidelines produced in these contexts will not necessarily be applicable in countries with different cultures and health systems. We therefore wished to explore the possibility of developing suicide first aid guidelines for a number of Asian countries. This project was undertaken to establish whether the use of the Delphi method is feasible in the development of suicide first aid consensus guidelines for these countries. This method was previously successfully implemented in the production of first aid guidelines for psychosis in Asia [15].

The aim of this project was to produce guidelines for use in particular Asian countries on how a member of the public should provide first aid to a person who is suicidal. The project did not aim to test hypotheses, rather to develop guidelines on first aid actions based on the consensus of expert clinicians. The project involved undertaking separate studies in three countries: Japan, Philippines and India. These three countries were chosen because they are Asian countries with different cultural and religious contexts, different rates of suicide, different levels of economic development, and different levels of availability of mental health services. We, therefore, expected that there would be differences across countries in the views expressed by the expert panels about appropriate guidelines for mental health first aid in relation to suicide [21-24]. This article presents the results of the study in Japan. The results from India and the Philippines have already been published [25,26]. To the best of the authors' knowledge, no study of this kind has been conducted in these countries before.

## Methods

The first aid guidelines were produced using a three-stage process: (a) a systematic search of the relevant evidence and claims made by authors of consumer and carer guides and websites; (b) the development of a questionnaire on possible first aid actions which was based on the search; (c) and the consensus of panels of clinicians from each of the countries on which first aid actions should be included in the guidelines.

### Systematic search for possible suicide first aid actions in the literature

As part of the project to develop suicide first aid guidelines for developed English-speaking countries [12], a systematic search for possible first aid actions was carried out in the formal professional literature listed in PubMed and PsycLit and other sources such as existing general mental health first aid manuals [7], other relevant manuals and guides on suicide prevention (e.g. Suicide Prevention Skills Training, [27]; Mental Health for Emergency Departments [28]) and relevant web sites (e.g. Samaritans).

### Construction of the questionnaire

A questionnaire was constructed from a content analysis of the actions indicated in the literature. Only statements that suggested a potential first aid action (i.e. what the first aider should do) or relevant awareness statements (what the first aider should know) were included in the questionnaire. These statements were grouped into common themes and used by a working group to generate questionnaire items specifying what actions a first aider should take. No judgments were made by the working group about the potential usefulness of the statements. Anything was included that fitted the definition of first aid, even if contradictory to other statements.

The questionnaire developed for English-speaking countries had 114 items, each describing a potential action that a first aider could do, which could be put to the panel for rating. These items covered the following broad areas: identification of suicide risk, assessing seriousness of suicide risk, initial assistance, talking with a suicidal person, no-suicide contracts, ensuring safety, confidentiality, and passing time during the crisis. The items are shown in Additional File 1. For the Asian guidelines, we added a few other items based on previous work on suicide prevention in Asian countries [22,29]. The initial questionnaire thus contained 138 first aid action items, plus 13 questions on participants' socio-demographics, experience/training, and opinions on suicide first aid. Open-ended questions to generate additional culturally-specific items were also included. Given that this was an exploratory project, we used English-language questionnaires, because the cost of doing it in the experts' native languages would have been prohibitive.

### Forming the expert panel

**Figure 1:**
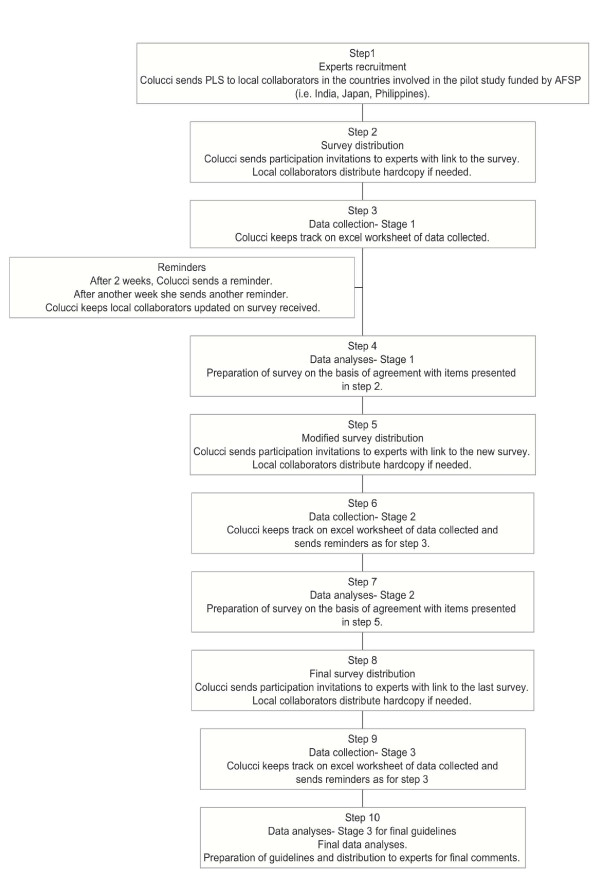
**Stages in the guidelines development**.

Professionals in Medicine, Public health, Social work and Psychology working in Japan were recruited by YS and EC (see Figure 1) to form the panel. Professionals recruited were actively involved in suicide prevention programs with the Ministry of Health, Labor and Welfare, or the Cabinet Office. The majority of the participants were local practitioners and some of them served in developing the Japanese national suicide prevention strategy.

In order to increase the cultural appropriateness of the guidelines, when forming the expert panels in each country we were careful to include as wide a representation (cultural and geographical) of professionals as possible.

When invitation letters, together with the Plain Language Statement, were sent to professionals asking them to be involved, they were also invited to nominate any colleagues who they felt would be appropriate panel members. During the recruitment process, potential participants were informed that one of the selection criteria was fluency in written English.

No attempt was made to make panels representative. The Delphi method does not require representative sampling; it requires panel members who are information and experience-rich [15].

The number of panel members in previous Delphi studies has varied considerably from 15 to 60 [30]. The presence of heterogeneous or homogeneous samples influences the decision of the appropriate number of participants. For a homogeneous group, 10 to 15 people can be sufficient, whereas heterogeneous group needs several hundred participants [31]. De Villiers and colleagues [32] argued that the number of participants can vary according to the purpose of the study, its complexity and resources, but a panel usually consists of 15 to 30 participants from the same discipline, or five to 10 per category from different professional groupings. The authors also observed that increasing the group size beyond 30 has seldom been found to improve the results. The participants in this study were all professionals with expertise in suicide prevention, therefore we aimed to have approximately 25-30 members in the panel.

### Delphi process

In Round 1 of the Delphi process, panel members were asked to complete an on-line questionnaire. This was administered using SurveyMonkey [33], with the option to complete it by email or paper mail if this was not possible (although no participant opted for this alternative). The questionnaire consisted of a list of first aid actions to rate. Only actions that are do-able by mental health first aiders were included in the list of items to be rated. Members of the expert panel were given the following instructions to guide their judgments:

"The following questionnaire asks about the best way a member of the public can help someone who is thinking about, or planning to suicide. Mental health first aid is defined as help given to someone who is experiencing a mental health problem, or is in a mental health-related crisis, until professional help is received or the crisis resolves. It does not include counseling or therapy. In the case of suicide, mental health first aid is given until the person decides to accept professional help, or decides against suicide. People who offer mental health first aid may be friends, family members, colleagues or acquaintances. They may or may not be involved in the person's life before or after offering first aid. For brevity, we will refer to the person offering assistance as "the first aider". When completing this questionnaire, you will read statements describing possible actions that the first aider can take to assist a suicidal individual. You will be asked to rate how important each item is as a guideline for a first aider. Please rate as "essential" or "important" those items which you feel should guide most people, most of the time, when assisting a suicidal person. The statements in this questionnaire were derived from a search of both professional and lay literature in English-speaking western countries. Therefore, there will be actions which would be appropriate for members of the public in your country which are not included and there will be actions that may be appropriate in Western countries but not in your cultural context. At the bottom of each page, there is room for you to add suggestions. Please consider the cultural, social and religious environment where you live, and try to add some relevant suggestions on each page. The more panel members add to this questionnaire, the more relevant and useful the guidelines will be for each individual country. Thank you for taking the time to assist us in this important suicide prevention project!".

The definition of mental health first aid given to the panel in the above instructions distinguishes a first aider's role from that of a clinician. In the case of a suicidal person, the first aider responds by getting professional help for the person, and supporting the person and ensuring their safety until the crisis has passed or until the suicidal person is receiving professional treatment and care. The guidelines needed to focus on the immediate prevention of suicide and not on solving the issues that led to the crisis.

Panel members were asked to rate each statement according to how important they believed it was as a potential first aid action for helping a suicidal person. The response scale was: 1. Essential; 2. Important; 3. Don't know/Depends; 4. Unimportant; 5. Should not be included. The scale was purposefully asymmetric because only items with positive ratings were of interest for the guidelines. This scale has worked well in previous guideline development work [12].

At the end of each block of items, the participants were asked to give any comments. In particular, they were invited to comment on items that were in the initial questionnaire that they considered to be culturally irrelevant or unacceptable, or that would not be feasible because of the local health system and other resources. Panel members were also invited to add any additional actions that were not included in the questionnaire. This was the place where culturally specific material could be introduced. The suggestions made by the panel members in response to the open-ended questions were reviewed by the research team and used to construct new items. Suggestions were accepted and added to Round 2 if they represented a new idea, could be interpreted unambiguously and were actions. Suggestions were rejected if they were near-duplicates of items in the questionnaire, if they were too specific, too general or were more appropriate to therapy than first aid.

One of the main advantages of using the Internet to collect data was that it made it easy for the survey manager to identify those participants who were late in completing the questionnaire or had completed only part of it and, thus, send reminders (with no need to send extra copies of the questionnaire, which can increase waiting times). Furthermore, no questions were inadvertently missed as the web survey was set up so that each question was mandatory. In addition, such survey software allows for branching (so that participants who did not endorse the use of no-suicide contracts were not asked to answer questions about what such a contract should contain).

In previous Delphi studies on suicide first aid, items were included in the guidelines if at least 80% of the panel rated them as "Essential" or "Important, and items were re-rated if 70-79% of the panel endorsed them. However, the research team made a decision to lower the threshold for the Japanese expert panel, because of the small number of items endorsed (mainly due to a large number of "Don't know/depends" answers). Thus, items rated as "Essential" or "Important" by 70% or more of the panel were included in the guidelines, and re-rated if there was 60-69% endorsement.

In Round 2, a second questionnaire was prepared. This consisted of items that met the 60-69% endorsement criterion for re-rating and new items that were generated from the comments in Round 1. In addition, a small number of items that received more than 50% of "Don't know/Depends" or "Not sure" answers were reworded, clarified or specified, and re-rated. In particular, following the comments made by a few participants, some of the items on the "no-suicide contracts" were reworded using the term "agreement" instead. Participants received an email with an individualized link to the online survey and a Word file that fed back a statistical summary of the items that were to be re-rated (i.e. their own original response to the item together with total percentages of endorsement of the item). They were told that they were able to change their responses when re-rating an item if they wished to do so. At the end of this round, any item that reached the 70% consensus criterion was selected for inclusion in the guidelines, any new item reaching the 60-69% consensus criterion was re-rated in Round 3, and the rest were rejected.

### Ethics

Ethics approval for the study was obtained from the University of Melbourne Human Research Ethics Committee (Project No. HREC 0605537).

## Results

### Sample

In Japan, 32 panel members were involved in Round 1 (i.e. 71% of the experts who were invited to participate), 28 in Round 2 and 26 in Round 3. All panel members were currently working in Japan. The majority were psychiatrists (N = 15) and psychologists (N = 5). The remaining were psychiatrist nurses and social workers. The panel was comprised of 18 males and 14 females. The majority of the participants were in the age range 30-39 years (N = 19) and 40-49 years (N = 5), four participants were aged 18-29 years, three were aged 50-59 years, and one was aged 60 years or over.

Some information was also collected on the clinical experience of the panel members. On average, participants reported that they had practiced in mental health/psychiatry for 12 years (the shortest time was 3 years and the longest 32 years). Just over a quarter of the participants (N = 9) received some formal education related to their profession overseas (mainly in USA). Slightly over a third of the participants (N = 12) reported having received formal training specifically on suicide prevention or intervention. However, when asked to state how well prepared they felt to assist a suicidal person, 16 answered "somewhat prepared", 12 "mostly prepared" and 2 "very prepared". Two participants reported being "not at all prepared". Although participants generally felt prepared to assist a suicidal person, in their opinion most people in Japan are not at all prepared (N = 19) or somewhat prepared (N = 12). Only one participant believed others are "mostly prepared" and no one believed that most people are "very prepared" to assist.

### Item endorsement

After three Delphi rounds, there were 56 items that were rated as "essential" or "important" by 70% or more of the panel members.

At Round 2, 38 new items suggested by participants were added to the questionnaire. The following are examples of such items:

• An important warning sign for suicide is if a person is saying they wish or intend to see or speak to someone who is dead (e.g., a deceased family member).

• An important warning sign for suicide is if a person is suddenly cleaning up their things (e.g. putting their room or desk in order).

• An important warning sign for suicide is if a person expresses in words or actions a sense of shame (e.g. from failure or loss).

• The first aider should be aware that suicidal people differ in their chosen suicide methods, so they should pay attention to the presence of any sort of potential suicidal means (not just guns, rope, pills but also knives, any kind of poison, kerosene and so on).

• The first aider should tell the person that they have done the right thing in talking about their suicidal thoughts.

• The first aider should try to convince the suicidal person that it is better to not keep their suicidal intentions a secret but involve someone else (e.g. a professional or a family member).

A number of responses to the Round 1 open-ended questions did not meet criteria for creation of a new item (e.g. they did not fit the definition of first aid or did not suggest a clear action) or were comments/suggestions. The following are examples (quoted verbatim, with minimal editing) of the comments and suggestions that did not generate new items but, nevertheless, are worthy of consideration:

• First Aid guidelines and the training program will not only reduce suicide, but also promote understanding of suicide and mental illness in public. In future, this First Aid should be taught to junior or high school student. I hope you will develop the guideline both in English and in our language so that we can use it.

• In our country (Japan), even mental health professionals are not always familiar with first-aid to suicide, and treatments vary from person to person. So the first step to disseminate first-aid actions should be started with the mental health professionals.

• In Japan, the social beliefs are somehow different from Western countries. Therefore, if we import the MHFA acts, we need to modify it.

• Thank you to request me to answer these questions. So interesting. We Japanese need mental health first aid for suicide, but in our culture/society this is a more ambiguous phrase, I think. I will at first stage focus on giving training to the possible first aider concerning building attitude to listen to others (not only suicidal persons) with empathy.

• The action and commitment of first aider seems to depend much on the background of first aiders (their position, profession and so on).

See the Additional File 1 for a complete list of rated statements, including the percentage of panel members endorsing each item.

At the end of the survey, participants were asked their opinions about the likely effectiveness of suicide first aid, using a 5-point Likert scale (from "definitely yes" to "definitely no"). Twenty-seven of the 29 participants who answered the question believed that if the first aider does the right thing, the risk of suicide can be reduced, whereas two answered "don't know/depends". The majority of the respondents (i.e. 19/29) thought that if the first aider does the wrong thing, the risk of suicide can be increased, whereas 7 answered "don't know/depends" and three "probably no".

The longer-term goal of the project is to use the guidelines to develop, implement and evaluate a training program on suicide first aid in Japan. This goal reflects the opinion of the expert panel. When asked if they thought members of the public should receive such training, 25 participants responded affirmatively. Only three respondents answered "don't know/depends" and one "probably no".

### Development of a guidelines document

The output from the Delphi process was a set of 56 agreed upon statements referring to actions that can be done by a mental health first aider. To be usefully communicated, this list of action statements was transformed into narrative text - the Suicide First Aid Guidelines (see Additional File 2).

The draft guidelines were sent to all panel members for their comments and final endorsement. Since the guidelines were meant to be useful to members of the public, it was important to ensure that they were written to be comprehensible to the target non-professional readership. Feedback from panel members was explicitly sought on the structure and readability of the guidelines, and suggested improvements were incorporated in the final version.

## Discussion

This project has demonstrated that it is possible to achieve consensus among mental health professionals on first aid strategies for suicidal thoughts and behaviour, and that the Delphi method is suitable for developing consensus guidelines in Japan. The method has been similarly successful in the Philippines [25] and India [26]. We would suggest that guideline development studies, using a similar method, could be carried in a number of other countries. As well as developing country-specific guidelines, it will also be possible to develop guidelines that are appropriate for cultural minorities within a country. This approach has been used in Australia, with a separate Delphi study undertaken to develop guidelines for Indigenous Australians, using Aboriginal mental health experts as panel members [34,35], and specific teaching programs have been developed for non-English speaking immigrant communities [36,37]. Inclusion and exclusion criteria, however, must consider cultural attitudes towards expressing opinions. For instance, while the threshold for items acceptance had to be lowered in the current study (from 80% to 70%) because of the large presence of "don't know/depends" answers, the opposite issue emerged in the study with Indigenous Australians where participants expressed agreement with the majority of items and the acceptance criteria was thus increased to 90%.

The Japanese experts expressed higher agreement on the item "The first aider should keep in mind that asking too many questions can provoke anxiety in the suicidal person" compared to Indian and Filipino experts. Furthermore, other items that could express a more direct or invasive involvement of the first aider were rejected in this country. Japan was also the only country where no statement in the section "Passing time" was agreed upon by the panel. Such findings raise the possibility that the role of a first aider might partially be culturally-bound and, for instance, in countries characterized by greater personal distance such as Japan, some behaviours that could be appropriate from the perspective of first aid strategies could be seen as socially inappropriate. Such differences should be further investigated and considered when developing training programs.

The next steps will be dissemination and use of the guidelines for the purpose of increasing community members' ability to recognize the risk of suicide and undertake basic first aid actions. These guidelines can be used as a source of advice to the public, as a basis for determining the curriculum of first aid training courses, and as a standard against which to evaluate the quality of existing materials and programs. The guidelines can inform the development of culturally appropriate training programs and information materials for how a member of the public can assist someone who is suicidal. At present there are limited structured training programs on suicide management skills available in Japan. Although much training is implemented throughout Japan, most of them mainly consist of didactic lectures with a focus on knowledge of facts about suicide. Provision of information alone may increase the knowledge of the audience; however, it does not necessary change attitudes or helping behaviour towards people with suicidal thoughts [38]. Training on skills enhancement based on these guidelines will certainly be beneficial for raising awareness and strengthening capacity at a community level, and preliminary work to further expand and develop a structured training program is currently underway. It will, of course, be necessary to evaluate the impact of such a training program [36,39,40].

This is the last of the three guidelines that were developed as part of this research project and it is relevant to observe that, despite the diversity of the countries involved, there was consensus on a core set of first aid items that were considered suitable for assisting a suicidal person, and there were other items that were consistently rejected across countries. Nevertheless, there were also differences between these three countries (such as on keeping the suicidal risk a secret in India, suggestion and/or acceptance of items concerning religion in the Philippines, and a greater personal distance expected by the first aider in Japan,) as there are differences with the guidelines for developed English-speaking countries [12]. This observation highlights the presence of a number of core basic first aid strategies that might have a cross-national and cultural validity and should form the basis of any training program, and the importance of developing culturally-specific guidelines with the help of local experts.

### Limitations

One limitation of this study is the relatively small number of panel members, although Delphi studies have been successfully run even with smaller groups and a size of 20-30 experts is common for this sort of study. Three quarters of the professionals approached agreed to participate. It is likely that some professionals refused participation because of limited proficiency in English, the language used throughout the research project. It is also possible that selecting participants who were fluent in English might have led to the exclusion of experts who were less exposed to the international literature and therefore held more traditional views on suicide.

Administering the questionnaire in English rather than in Japanese limits the general applicability of the findings. It is possible that there would have been more culturally specific responses if panelists had used their native language.

Future studies should recruit broader and more representative expert panels including, where possible, professionals from all the relevant mental health disciplines and consumer and carer representatives. Participation of consumers and carers in such research, as members of an 'expert panel' rather than as research subjects, is still an uncommon practice [15], which also requires additional funding that was not available for the current project. Nevertheless, the local collaborator in this study (YS) and her team are planning to also have consultations with carers and people who have made a suicide attempt.

Another limitation is that the inclusion of culturally relevant material was dependent on panelists responding to the open-ended questions and not every participant did this. In some cases, this may have been due to lack of time or because the participants in this study were better able to read English than to write it. It may also be because, in questionnaires of all kinds that require ratings to be made, respondents rarely take the opportunity to write comments or to make suggestions when the opportunity is given [15]. However, it must be noted that, compared to other similar mental health first aid guideline research, a considerable number of suggestions for new items were given in this study. This might have been because of the emphasis in the instructions to participants in each section of the questionnaire to provide suggestions based on their cultural, social and religious settings.

Finally, while these guidelines have been developed for Japan, it is recognised that this country is characterised by diversity, for instance between rural and urban areas, and some prefectures are more affected by suicide than others.

## Conclusions

There is a growing awareness of suicide as a major public health problem, even though there is a taboo in many societies against discussing it openly [1]. Developing suicide first aid guidelines for community members, and training programs based on these, might also contribute towards changing society's attitudes towards suicide and towards those members of the community who are dealing with suicidal intentions.

This study has demonstrated that it is possible to reach consensus on the development of guidelines for Japan. Although the guidelines were designed for the public, they may also contain advice that might be helpful to people working in health and welfare professions.

Where the guidelines are used as the basis for first aid training, efforts need to be made to evaluate their impact on the first aider's helping behaviour and on the recipients of the first aid. This will assist researchers to develop an evidence base for mental health first aid and suicide prevention initiatives.

In a WHO news release [2] it was stated that "It's important to realise that suicide is preventable". By collaborating with local experts to agree on a minimum set of suicide first aid actions, and by making such guidelines freely and easily accessible to everyone, we hope to convey the message that suicide is preventable, suicide is everyone's business, and everyone can contribute to its reduction. Members of the general public have a crucial role to play in suicide prevention. Creating opportunities for the public to learn basic first aid actions, and how to implement them when needed, are important steps towards more effective and global suicide prevention activities.

## Competing interests

The authors declare that they have no competing interests.

## Authors' contributions

EC recruited clinical experts, revised the existing questionnaire, prepared and administered the on-line surveys for Round 1-3, analysed the data, prepared the draft and final guidelines and wrote the first draft of the manuscript. CMK wrote the former questionnaire and contributed to the revised version, supervised every stage of the project and co-wrote the guidelines. HM co-wrote the grant application, recruited the main local collaborators and clinical experts, and reviewed the Round 1 questionnaire and the guidelines. AFJ co-wrote the grant application and developed the method. YS was the main local collaborator in the study and recruited the majority of the clinical experts. All authors contributed to the writing of the manuscript and approved the final version.

## Supplementary Material

Additional file 1 **Table of data showing the items included in the Delphi survey and the endorsement levels from the Japanese panel members**. Click here for file

Additional file 2 **First aid guidelines for Japan**. This file may be distributed freely, with the authorship and copyright details intact. Please do not alter the text or remove the authorship and copyright details. Click here for file
